# Co-Application of 24-Epibrassinolide and Titanium Oxide Nanoparticles Promotes *Pleioblastus pygmaeus* Plant Tolerance to Cu and Cd Toxicity by Increasing Antioxidant Activity and Photosynthetic Capacity and Reducing Heavy Metal Accumulation and Translocation

**DOI:** 10.3390/antiox11030451

**Published:** 2022-02-24

**Authors:** Abolghassem Emamverdian, Yulong Ding, James Barker, Guohua Liu, Mirza Hasanuzzaman, Yang Li, Muthusamy Ramakrishnan, Farzad Mokhberdoran

**Affiliations:** 1 Co-Innovation Center for Sustainable Forestry in Southern China, Nanjing Forestry University, Nanjing 210037, China; ylding@njfu.com.cn (Y.D.); ramky@njfu.edu.cn (M.R.); mfarzad649@hotmail.com (F.M.); 2 Bamboo Research Institute, Nanjing Forestry University, Nanjing 210037, China; 3 School of Life Sciences, Pharmacy and Chemistry, Kingston University, Kingston-upon-Thames, Surrey KT1 2EE, UK; j.barker@kingston.ac.uk; 4 Department of Agronomy, Faculty of Agriculture, Sher-e-Bangla Agricultural University, Dhaka 1207, Bangladesh; 5 Department of Mathematical Sciences, Florida Atlantic University, Boca Raton, FL 33431, USA; yangli@fau.edu

**Keywords:** toxic metals/metalloid, nanoparticles, phytohormones, phytoremediation, reactive oxygen species

## Abstract

The integrated application of nanoparticles and phytohormones was explored in this study as a potentially eco-friendly remediation strategy to mitigate heavy metal toxicity in a bamboo species (*Pleioblastus pygmaeus*) by utilizing titanium oxide nanoparticles (TiO_2_-NPs) and 24-epibrassinolide (EBL). Hence, an in vitro experiment was performed to evaluate the role of 100 µM TiO_2_ NPs and 10^−8^ M 24-epibrassinolide individually and in combination under 100 µM Cu and Cd in a completely randomized design using four replicates. Whereas 100 µM of Cu and Cd reduced antioxidant activity, photosynthetic capacity, plant tolerance, and ultimately plant growth, the co-application of 100 µM TiO_2_ NPs and 10^−8^ M EBL+ heavy metals (Cu and Cd) resulted in a significant increase in plant antioxidant activity (85%), nonenzymatic antioxidant activities (47%), photosynthetic pigments (43%), fluorescence parameters (68%), plant growth (39%), and plant tolerance (41%) and a significant reduction in the contents of malondialdehyde (45%), hydrogen peroxide (36%), superoxide radical (62%), and soluble protein (28%), as well as the percentage of electrolyte leakage (49%), relative to the control. Moreover, heavy metal accumulation and translocation were reduced by TiO_2_ NPs and EBL individually and in combination, which could improve bamboo plant tolerance.

## 1. Introduction

In recent decades, increasing anthropogenic activities have led to increases in greenhouse gases in the natural environment, and chemical fertilization has led to increases in heavy metal contamination in forestland and agricultural soils, deleteriously contributing to global climate change [1]. Many reports have shown that heavy metals pose a major threat to agricultural land, animals, and plants, which can influence the human food chain, leading to negative effects on human health [2,3]. Copper (Cu) and cadmium (Cd) have been mentioned as being the most abundant toxic metals in Chinese farmland soils [4]. While there is no evidence of the biological activity of Cd in the plant growth process, trace amounts of Cu could have a positive impact as a dietary nutrient on plant growth; however, extreme Cu levels induce plant toxicity [5]. Cu, as a trace element, can enhance photosynthetic efficiency, such as electron transport. Additionally, Cu regulates structural proteins involved in cell wall metabolism and can elevate mitochondrial respiration to produce energy [6]. Conversely, surplus concentrations of Cu in the form of Cu^2+^ are responsible for oxidative stress due to the generation of reactive oxygen species (ROS) compounds [7], which can result in a reduction in plant growth with an altered functionality of the cell membrane, limitation of enzyme activities, and depression of photosynthetic efficiency, ultimately leading to plant death [8]. Cd, a nonessential element with high toxicity, is known as the most dispersed element in soils and irrigation water, which has a destructive influence on plant and human life [9]. Excess Cd in soil and the absorption of cadmium by plants increase ROS production, such as free radicals, which are the main factors in the initiation of oxidative stress in plants [9]. Cadmium has a damaging impact on plant cell functions and the metabolic pathways involved in the production of lipids, proteins, and nucleic acids. Cd injures the cell membrane, which leads to lipoperoxidation and oxidative toxicity in plants. It has been demonstrated that cadmium reduces the plant defense system with a reduction in antioxidant activity capacity. This phenomenon finally reduces plant photosynthesis and inhibits plant growth and development [9].

Nanoparticles with unique structures and sizes (1 to 100 nm) [10] have been observed to increase plant nutrients and crop production [11]. The diverse surface-to-volume ratios of nanoparticles could differ from their bulk counterparts [12]. Recently, some studies have reported that titanium nanoparticles (TiO_2_) have the ability to increase plant growth under metal stress [13,14,15,16,17]. Therefore, we suggest that titanium could be a good material to reduce plant stress. Brassinosteroids (BRs) are a new phytohormone and belong to the polyhydroxy steroidal group. There are 70 types of BRs in plants. Among them, 24-epibrassinolide (EBL) is known as the top bioactive BR that can promote plant growth under stressful conditions [18]. In addition, 24-epibrassinolide EBL induces antioxidant activity, plant photosynthesis, seed yield, and oxidative production under stressful conditions [19,20,21]. It has been indicated that the interaction of EBL with other cellular molecules can enhance signaling efficiency within the plant defense grid under stress conditions [22]. This phenomenon can boost antioxidant capacity in the face of multiple stressful factors, such as HMs [23]. This research study represents the individual and co-application of TiO_2_-NPs and EBL, as well as the investigation of their role in the alleviation of Cu and Cd toxicity in bamboo plants with an emphasis on antioxidant, photosynthetic, and plant growth parameters.

Bamboo (*Bambusoideae*) species occupy the largest portion of Chinese farmland (6 million hectares) [24,25]. This fast-growing plant provides nutrient sources for local family livelihoods in southern and western China [26]. *Pleioblastus pygmaeus* is a suitable species for landscape purposes, with a characteristic height of 30–50 cm. *Pleioblastus pygmaeus* originated in Japan but was transferred to China in the early 20th century. A desirable condition of this plant for this experiment was its adaptation to basic (alkaline), acidic, and neutral soils [27]. Conversely, the excess of heavy metals (frequently Cu and Cd) caused by anthropogenic activities has become a major dilemma for agricultural and forestry soils in this area [4], which can influence bamboo plant growth and development. Hence, it is essential to find appropriate biologic materials to reduce soil toxicity and increase plant tolerance under heavy metal toxicity. Therefore, we selected two applications of TiO_2_ NPs and 24-epibrassinolide, individually and in combination, against heavy metal toxicity, which could aid in understanding the involved mechanisms in the combined application of nanoparticles and phytohormones against heavy metal toxicity. To our knowledge, this is the first comprehensive study to investigate the combination of TiO_2_ NPs and EBL in the amelioration of Cu and Cd toxicity in bamboo species. Therefore, in this paper, we aim to investigate the impact of TiO_2_ NPs and EBL on enhancing plant tolerance under heavy metal toxicity with an emphasis on antioxidant and nonantioxidant enzyme capacity, ROS production, photosynthesis, and growth indices under Cu and Cd.

## 2. Materials and Methods

### 2.1. Plant Material and In Vitro Conditions

This research study was performed under in vitro conditions in a plant tissue culture laboratory using MS medium (Murashige and Skoog, 1962) [28] consisting of 6-benzylaminopurine (6-BA) (4 mL), micronutrients (10 mL), macronutrients (100 mL), kinetin (KT) (0.5 mL), sucrose (30 g) and agar (8 g) at pH 5.8 ± 0.1. For this purpose, a completely randomized design (CRD) was employed that contained 100 µM TiO_2_ NPs and 10^−8^ M 24 epibrassinolide individually and in combination with 100 µM Cu as well as 100 µM Cd in four replications (Table 1). We adjusted the pH value in MS to 5.8 for two reasons: firstly, to optimize nutrient absorption, the availability of the nutrients to the plants was optimum at pH 5.8; and secondly, the preparation of the gelling of the agar-solidified medium should be completed at ca. pH 5.8. To proliferate bamboo roots, young shoots (10 mm long nodal explants) were planted in MS medium supplemented with pyridoxine (3 μM), nicotinic acid (4 μM), thiamine–HCl (1.2 μM), myo-inositol (0.6 mM), 30 *g* L^−1^ sucrose, and 0.1 mg L^−1^ indole-3-acetic acid (IAA) as a regulator hormone involved in plant growth. The appropriate amount of each treatment (100 µM TiO_2_ NPs and 10^−8^ M 24-epibrassinolide) was mixed in 1 L MS medium, adjusted to pH 5.8 ± 0.1, and then applied to 8–10 *g*/L agar. The solution was placed in 60 mm diameter glass petri dishes containing 100 mL of culture, and sterilization of the intended MS medium was conducted in an autoclave (HiClave HVE-50, ZEALWAY-USA, Delaware, DE, USA) at the optimum temperature of 110 °C for 40 min. The dishes were transferred to an Air Tech incubation hood with ultraviolet sterilization with white fluorescent lamps (wavelength between 10 and 420 nm) at a temperature of 25 °C for 4 h. In the final step, the plantlet treatments were preserved as research materials in a controlled tissue culture chamber with fluorescent lamps (white) at a wavelength between 10 and 420 nm. In terms of temperature, the growth was performed at 17/22 °C in the dark periods and 30/25 °C in the light periods for three weeks.

Titanium nanoparticles were provided by Nanjing Jiancheng Company, Jiangsu Province, China, and consisted of a white powder with a purity of >99% nanotitanium and a diameter of 25 nm. The levels of Cu and Cd were chosen according to the previous studies, which displayed high and low levels of toxicity in bamboo plants [13,14]. Bamboo (*A. pygmaeus*) was selected from local species by the Bamboo Research Institute, which is located at Nanjing Forestry University.

In this research study, biomass and growth indices, including root and shoot dry weight (DW) and shoot length, were quantified. To investigate photosynthesis pigments, total chlorophyll (Chl), Chl a and b, and carotenoid contents were measured. To determine the fluorescence parameters, 5 parameters were recorded, including: (i) actual photochemical efficiency of PSll (φPSll), (ii) maximum photochemical efficiency of PSll (Fv/Fm), (iii) photochemical quenching coefficient (qP), (iv) effective photochemical efficiency of PSll (Fv′/Fm′), and (v) nonphotochemical quenching (NPQ). Heavy metal accumulation and TiO_2_ NP contents were measured in leaves, stems, and roots. Plant defense enzymes and nonenzymatic antioxidants were measured. To assay cell membrane injury, ROS compounds, electron leakage, and malondialdehyde (MDA) content were estimated. Finally, the translocation factor (TF), bioaccumulation factor (BAF), and tolerance index (TI) of the shoots and roots were calculated.

### 2.2. Preparation of Samples

Leaf samples were collected from the different treatments, and then 0.5 g samples were placed in a container and crushed into a powder. An appropriate amount of liquid nitrogen was added to the samples, and the obtained powder was dissolved in PBS (pH 7.2–7.4) at 2–8 °C. The solution was centrifuged at 2500–3500× *g* for 17 min to extract the supernatant, which was kept for use in antioxidant enzyme activity tests.

### 2.3. Protective Enzymes

Superoxide dismutase (SOD, EC: 1.15.1.1) was measured based on the results of photoreduction obtained by nitro blue tetrazolium (NBT), which was conducted using the Zhang method [29]. Peroxidase (POX, EC: 1.11.1.7) was estimated by using the protocol of Upadhyaya [30]. Catalase (CAT, EC: 1.11.1.6) was estimated based on the results of reactions analyzing H_2_O_2_ at an absorbance of 240 nm, which was estimated by the Aebi protocol [31]. Glutathione reductase (GR, EC: 1.6.4.2) was estimated using the protocol reported by Foyer and Halliwell [32] with some modifications. Ascorbate peroxidase (APX, EC: 1.11.1.11) was measured using the Nakano and Asada method [33]. APX antioxidant activity was obtained by recording the reduction in absorbance at 290 nm (coefficient of absorbance at 2.8 mM^−1^ cm^−1^). Phenylalanine ammonia-lyase (PAL, EC: 4.3.1.5) activity was assessed using the Berner [34] protocol.

### 2.4. Assessment of Nonenzymatic Antioxidant Activities (Flavonols, Tocopherols, and Total Phenolics)

#### Methanolic Extract Preparation

For this test, 0.5 g of dry leaf sample was dissolved in 4 mL of methanol (80%) and then centrifuged at 7000× *g* for 15 min. The methanolic extract was used for the tests. The total phenolics were measured according to the protocol of Conde [35]. According to this protocol, a 0.1-m methanolic extra was added to 2.5 mL of 10% Folin–Ciocalteu reagent. Then, for neutralization of the obtained mixture of sodium bicarbonate, 7% was added. The final mixture was transferred to a spectrometer machine to measure the total phenolics at an absorbance of 765 nm. The content of flavonol was determined according to the Akkol method [36]. A 0.5-mL methanolic extract was homogenized with 0.4 mL of aluminum chloride (2%) and 1.5 mL of sodium acetate (5%). After preparation of the supernatant, it was kept at room temperature for 2.5 h. The flavonoid content was determined in the supernatant at an absorbance of 445 nm. The content of tocopherol was determined according to the protocol of Kayden [37]. For this purpose, 3 mL ethanol was mixed with 0.1 g of leaf samples, and the soluble solution was then centrifuged at 7000× *g* for 15 min. The obtained mixture was added to 0.1 mL ethanol extract, 0.2 mL bathophenanthroline at a concentration of 0.2%, 0.001 M of 0.2 mL ferric chloride, and 1 mM 0.2 mL phosphoric acid. The content of tocopherol was recorded by measuring the absorbance of the supernatant at 534 nm.

### 2.5. Assay of Hydrogen Peroxide (H_2_O_2_), Malondialdehyde (MDA), Superoxide Radical (O_2_^•−^), Soluble Proteins (SP), and Electrolyte Leakage (EL)

Malondialdehyde is representative of lipid peroxidation, which was measured by the protocol described by Siddiqui [38]. In this experiment, 0.1% trichloroacetic acid (TCA) was used for the homogenization of fresh leaves, after which the sample was centrifuged at 8000× *g* for 25 min. The obtained amount of supernatant was mixed with TCA solution in the range of 20%, which contained 0.5% thiobarbituric acid. In the next process, the soluble solution was kept at 98 °C for 25 min. Then, the soluble solution was kept at room temperature. The final soluble solution was centrifuged a second time at 2000× *g* for 15 min at 5 °C. Finally, to estimate malondialdehyde, the absorbance was determined at 532 nm.

The levels of H_2_O_2_ were determined using the protocol reported by Patterson [39]. For this study, samples (leaves) in the specified amount of 0.5 g were mixed in a mortar and pestle by adding 10 mL cold acetone. The mixture was centrifuged at 4000× *g* for 25 min. In the next step, titanium chloride at a concentration of 20% in 2 mL of concentrated HCl and 2 mL of ammonia at the specified level of 17 M were added to the supernatant (1 mL). The supernatant was extracted with acetone, which was conducted by the addition of 2 N H_2_SO_4_ in 10 mL for proper absorbance. To remove immiscible inputs, the mixture was centrifuged again. The absorbance of the supernatant was recorded at 410 nm. The levels of H_2_O_2_ were determined based on a standard curve, which was created based on the known levels of H_2_O_2_ and formulated as μmole g^−1^ FM. The soluble protein (SP) levels were assigned according to the protocol of Bradford [40] and measured based on the effect of Coomassie Brilliant Blue (G25) on changes in protein levels. The final data were obtained using a spectrometer machine. The amount of superoxide radical (O_2_^•−^) was determined according to the method of Li [41]. According to this protocol, 200 mg leaf tissue samples were mixed with phosphate buffer at pH 7.8 in the amount of 65 mM and then centrifuged at 4000× *g* for 20 min. The supernatant was incubated in 10 mM of hydroxylamine hydrochloride and 65 mM of phosphate buffer (pH = 7.8) for 15 min at 27 °C. In the next step, 7 mM α-naphthylamine plus 17 mM sulfanilamide was added to the mixture, preserved for 25 min and then recorded at an absorbance of 530 nm at 25 °C. Finally, to determine the final rate of O_2_^•−^, nitrogen dioxide radicals (NO_2_) were applied to generate a standard curve. Electrolyte leakage (EL) was calculated based on the protocol of Valentovic [42]. According to this protocol, 0.3 g of leaf samples were mixed with 15 mL of deionized water. Then, the mixture was kept at the optimum temperature (25 °C) for 2.5 h. In this stage, EC_1_ was recorded as the primary electrical conductivity of the mixture. To obtain EC_2_ as the secondary electrical conductivity, the samples were transferred to one autoclave and kept at 120 °C for 17 min. At the end of the test, EL was determined based on the following formula:

EL (%) = EC_1_/EC_2_ × 100 (1)

### 2.6. Measurement of Photosynthetic Pigments and Fluorescence Parameters

Photosynthetic pigments, such as Chl a and b, and carotenoid levels were determined according to the protocol of Lichtenthaler and Buschmann [43]. For pre-experiment bamboo samples, ca. 0.5 g was provided, and then the samples were transferred to a mortar with liquid nitrogen. To prepare the liquid sample extract, the obtained powder was mixed with 20 mL of acetone at a specific concentration of 80% at 0 to 5 °C. Then, it was centrifuged at 5000× *g* for 15 min. At the end of the experiment, Chl a, Chl b, and carotenoid levels were determined at absorbances of 663, 645, and 470 nm, respectively. Finally, the levels of Chl and carotenoids were calculated based on the following formulae, which were set in units equal to mg/*g* fresh weight:

Chlorophyll a = 12.25A663 − 2.79A647(2)

Chlorophyll b = 21.50A647 − 5.10A663(3)

Total Chl = Chl a + Chl b(4)

Carotenoid = 1000A470 − 1.82Chl a − 95.15 Chl b/225(5)

A chlorophyll fluorescence imager (CFI) (England) was used to measure fluorescence characteristics, which was conducted under specific dark-adapted conditions for 35 min. To measure the light fluorescence parameters, the fluorescence characteristics were recorded at 700 micromoles m^−2^ s^−0^ in an illumination incubator for actinic light activation. In this study, the main fluorescence indices were: (i) actual photochemical efficiency of PSll (ɸPSll); (ii) effective photochemical efficiency of PSll (Fv′/Fm′); (iii) photochemical quenching coefficient (qP); (4) maximum photochemical efficiency of PSll (Fv/Fm); and (5) non-photochemical quenching (NPQ).

### 2.7. Measurement of TiO_2_ NPs and Metal Accumulation in Roots, Stems, and Leaves of Bamboo

Copper (Cu) and cadmium (Cd) contents and titanium accumulation in roots, stems, and leaves of bamboo species were investigated. For their measurement, the samples were cleaned and dried in an oven, and subsequently 70% nitric acid was added to the samples, which were preserved at an optimum temperature of 70 °C for 20 min. Then, the obtained solution was centrifuged at 9000× *g* for 20 min. The contents of Cu and Cd in the plant organs, such as roots, stems, and leaves, were assigned and analyzed using an atomic absorption spectrometry machine (AAS HITACHI, High-Tech Company Tokyo, Japan). In this process, a spectrometer equipped with a furnace of graphite and correction system of the Zeeman-effect background was performed (AAnalyst 800, Perkin Elmer, Norwalk, CT, USA). For determination of the element contents, the different characteristics of the instruments were adjusted. Standardization of the metal was performed using 2.5% nitric acid (spectra scan). For calibration, confirmation of the standard (Perkin Elmer), which contained all of the elements in one inorganic target analyst list (TAL), was performed at optimum intervals in one unattended automatic analysis run.

### 2.8. Biomass Determination and Shoot Length

Plant biomass was determined by measuring the dry weight of roots and shoots. Firstly, samples (root and shoot treatment) were cleaned and then placed in an oven to remove the water from their surfaces during the process. The samples were fixed at 115 °C for 25 min. To determine the plant dry weight, the samples were maintained at 70 °C for 10 h and then weighed. To determine the shoot length, bamboo plants were measured at the beginning and end of the study. The final data were defined as the difference in plant height.

### 2.9. Determination of the Tolerance Index (TI), Bioaccumulation Factor (BAF), and Translocation Factor (TF)

To determine the plant tolerance to Cu and Cd toxicity, the indices of the tolerance index (TI), bioaccumulation factor (BAF), and translocation factor (TF) were calculated. This result was obtained based on the method of Souri and Karimi [44] and is considered to denote the efficiency of photoextraction. The formula below is used to find the value of TI, which represents the tolerance index in the shoot and root; TF, which represents the translocation factor; and BAF, which represents the bioaccumulation factor of leaves, stem, and root.

TF (leaves/stem) = the concentration of the co-application of Ti–EBL/heavy metals (Cu, Cd) in the leaves/stem of plants (mg/kg)/the concentration of the co-application of Ti–EBL/heavy metals (Cu, Cd) in the roots of plants(6)

TI shoot/root = the dry weight of plant shoot/root from the co-application of Ti–EBL/heavy metal (Cu, Cd) treatment (g)/the dry weight of plant shoot/root from the control (g)(7)

BF (leaves/stem/root) = concentrations of heavy metals in the leaves or stem or root/concentrations of heavy metals in the medium(8)

### 2.10. Statistical Analysis

A completely randomized design (CRD) was used in this study, consisting of a 2-way factorial with four replicates. R software was used for ANOVA (analysis of variance), and the Tukey’s test was used to determine mean differences, which were conducted at the *p* < 0.05 probability level.

## 3. Results

### 3.1. 24-Epibrasinolide and Titanium Oxide Nanoparticles Promote Antioxidant Capacity in Plants under Cu and Cd Toxicity

**Figure 1:**
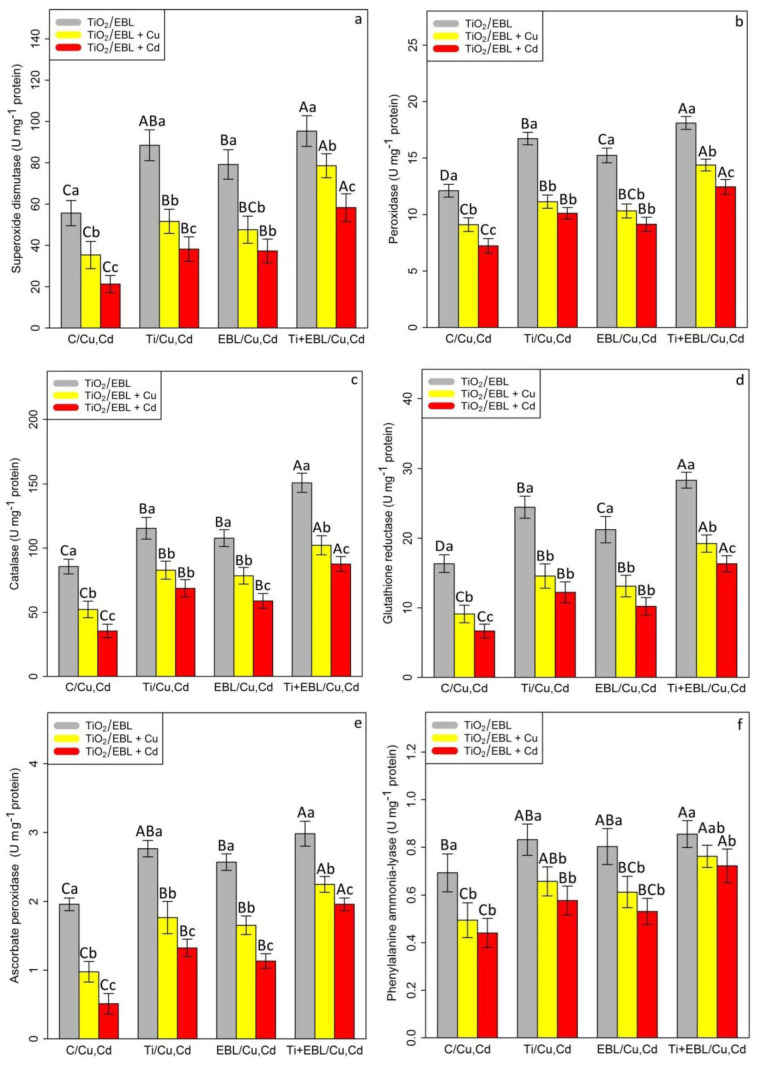
The impact of the co-application of 24-epibrassinolide and titanium oxide nanoparticles individually and combined on antioxidant enzyme activities (superoxide dismutase (SOD) (**a**), peroxidase (POX) (**b**), catalase (CAT) (**c**), glutathione reductase (GR) (**d**), ascorbate peroxidase (APX) (**e**), and phenylalanine ammonia-lyase (PAL) (**f**)) in bamboo species (*Pleioblastus pygmaeus*) with 100 μM Cu and 100 μM Cd. In this study, 1-year-old branches of *P. pygmaeus* were used as plant treatments together with 100 µM TiO_2_ NPs and 10^−8^ M 24-epibrassinolide, individually and in combination with 100 µM Cu and 100 µM Cd using four replications. Planting of the treated bamboo was performed in an Air Tech inoculation hood with fluorescent white lamps and ultraviolet light (wavelengths of 10–400 nm) at 15 °C and 30 °C. The bamboo plants were constantly exposed to excess heavy metals for three weeks. Sampling for the measurement of antioxidant enzyme activity (**a**–**f**) was conducted after three weeks of plant exposure to the co-application of 24-epibrassinolide and titanium oxide nanoparticles under 100 µM Cu and 100 µM Cd. The capital letters (^A–C^) indicate significant differences between treatments of control (C), titanium (Ti), 24-epibrassinolide (EBL), and 24-epibrassinolide involving individual or combined application of titanium oxide nanoparticles (EBL–TiO_2_ NPs) under 100 μM Cu and 100 μM Cd (the bars with similar colors), while the lowercase letters (^a–c^) denote statistically significant differences at each concentration of the co-application of EBL and TiO_2_ NPs, individually or in combination with 100 µM Cu and 100 µM Cd (the bars with various colors) based on Tukey′s test (*p* < 0.05).

The data analysis revealed a significant difference between the various treatments of the co-application of EBL–TiO_2_ NPs with Cu and Cd (*p* < 0.001). According to the obtained data, TiO_2_ and EBL individually could increase antioxidant activity under stress conditions. However, the greatest stimulation of antioxidant activity was observed with the combination of TiO_2_ and EBL. The combination of TiO_2_ and EBL showed the highest capacity for antioxidant activity stimulation, with a 1.71-fold enhancement of SOD, 1.49-fold increase in POX, 1.76-fold increase in CAT, 1.52-fold increase in APX, 1.73-fold enhancement of GR, and 1.23-fold enhancement of PAL activity in comparison with their control treatments (Figure 1). Conversely, the lowest amount of antioxidant activity was observed with 100 µM Cu and 100 µM Cd, which resulted in 36% and 61% reductions in SOD, 24% and 40% reductions in POX, 39% and 58% reductions in CAT, 50% and 73% reductions in APX, 44% and 59% reductions in GR, and 28% and 36% reductions in PAL activities, respectively, compared with the control treatment. According to these results, we suggest that TiO_2_ and EBL individually have the potential to reduce Cu and Cd toxicity, but the combination of TiO_2_ and EBL has a larger impact on the amelioration of heavy metal toxicity.

### 3.2. 24-Epibrasinolide and Titanium Oxide Nanoparticles Reduce Malondialdehyde (MDA), Soluble Proteins (SP), Electrolyte Leakage (EL), Hydrogen Peroxide (H_2_O_2_), and Superoxide Radicals (O_2_^•−^)

**Figure 2:**
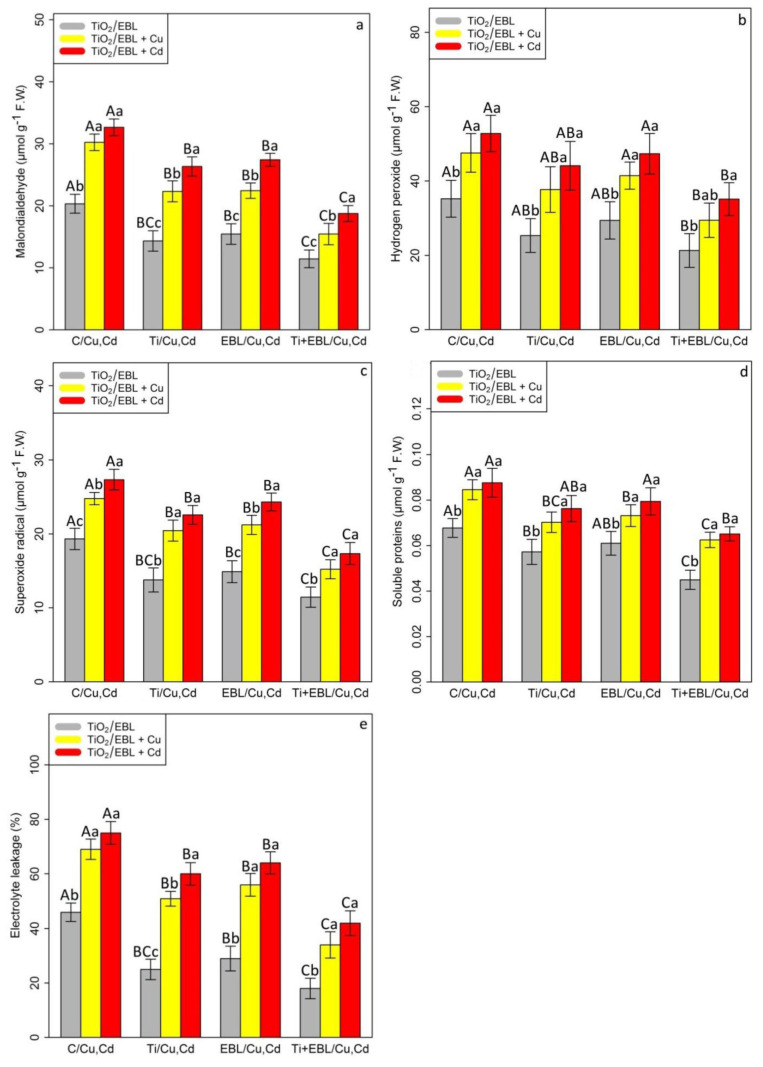
The impact of the co-application of 24-epibrassinolide and titanium oxide nanoparticles individually and combined on malondialdehyde content (MDA) (**a**), hydrogen peroxide (H_2_O_2_) (**b**), superoxide radical (O_2_^•−^) (**c**), soluble proteins (SP) (**d**), and electrolyte leakage (EL) (**e**) in bamboo species (*Pleioblastus pygmaeus*) with 100 μM Cu and 100 μM Cd. In this study, 1-year-old branches of *P. pygmaeus* were used as plant treatments together with 100 µM TiO_2_ NPs and 10^−8^ M 24-epibrassinolide, individually and in combination with 100 µM Cu and 100 µM Cd using four replications. Planting of the treated bamboo was performed in an Air Tech inoculation hood with fluorescent white lamps and ultraviolet light (wavelengths of 10–400 nm) at 15 °C and 30 °C. The bamboo plants were constantly exposed to excess heavy metals for three weeks. Sampling for the measurement of MDA, H_2_O_2_, O_2_^•−^, SP, and EL (**a**–**e**) was conducted after three weeks of plant exposure to the co-application of 24-epibrassinolide and titanium oxide nanoparticles under 100 µM Cu and 100 µM Cd. The capital letters (^A–C^) indicate significant differences between treatments of control (C), titanium (Ti), 24-epibrassinolide (EBL), and 24-epibrassinolide involving individual or combined application of titanium oxide nanoparticles (EBL–TiO_2_ NPs) under 100 μM Cu and 100 μM Cd (the bars with similar colors), while the lowercase letters (^a–c^) denote statistically significant differences at each concentration of the co-application of EBL and TiO_2_ NPs, individually or in combination with 100 µM Cu and 100 µM Cd (the bars with various colors) based on Tukey′s test (*p* < 0.05).

Our results showed that the TiO_2_ NP and EBL concentrations had the ability to reduce ROS compounds and prevent cell membrane injury. The data analyses showed a significant difference in the co-application of TiO_2_ NPs and EBL based on the concentration of Cu and Cd in the indices of MDA, H_2_O_2_, SP, EL, and O_2_^•−^ (*p* < 0.001). The most positive effect of the treatments on heavy metals was related to the combination of TiO_2_–EBL with Cu and TiO_2_–EBL with Cd, which demonstrated 49% and 42% reductions in MDA content, 38% and 33% reductions in H_2_O_2_ content, 38% and 36% reductions in O_2_^•−^ content, 26% and 25% reductions in SP content, and 50% and 44% reductions in EL, respectively. Additionally, the levels of TiO_2_ and EBL individually showed a positive role in the amelioration of oxidative stress, which occurred by restraining ROS production, which in turn resulted in protecting the cell membrane against oxidative free radicals. Conversely, the results showed that 100 µM Cu and 100 µM Cd increased the levels of MDA, H_2_O_2_, O_2_^•−^, SP, and EL, with 48% and 60% increases in MDA content, 35% and 49% increases in H_2_O_2_, 28% and 41% increases in O_2_^•−^, 25% and 29% increases in SP, and 50% and 63% increases in EL, respectively, compared with their control treatments (Figure 2).

### 3.3. 24-Epibrasinolide and Titanium Oxide Nanoparticles Increase Nonenzymatic Antioxidant Activities (Flavonol, Tocopherol, and Total Phenolics) in Bamboo Species under Cu and Cd Toxicity

**Figure 3:**
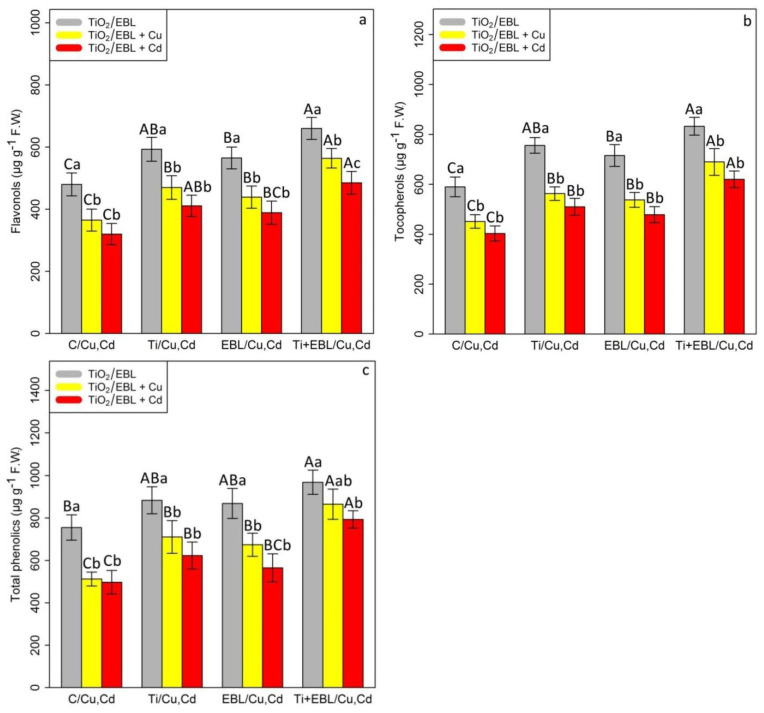
The impact of the co-application of 24-epibrassinolide and titanium oxide nanoparticles individually and combined on nonenzymatic antioxidant activities ((**a**) flavonols, (**b**) tocopherols, (**c**) total phenolics) in bamboo species (*Pleioblastus pygmaeus*) with 100 μM Cu and 100 μM Cd. In this study, 1-year-old branches of P. *pygmaeus* were used as plant treatments together with 100 µM TiO_2_ NPs and 10^−8^ M 24-epibrassinolide, individually and in combination with 100 µM Cu and 100 µM Cd, using four replications. Planting of the treated bamboo was performed in an Air Tech inoculation hood with fluorescent white lamps and ultraviolet light (wavelengths of 10–400 nm) at 15 °C and 30 °C. The bamboo plants were constantly exposed to excess heavy metals for three weeks. Sampling for the measurement of flavonols, tocopherols, and total phenolics (**a**–**c**) was conducted after three weeks of plant exposure to the co-application of 24-epibrassinolide and titanium oxide nanoparticles under 100 µM Cu and 100 µM Cd. The capital letters (^A–C^) indicate significant differences between treatments of control (C), titanium (Ti), 24-epibrassinolide (EBL), and 24-epibrassinolide involving individual or combined application of titanium oxide nanoparticles (EBL–TiO_2_ NPs) under 100 μM Cu and 100 μM Cd (the bars with similar colors), while the lowercase letters (^a,b^) denote statistically significant differences at each concentration of the co-application of EBL and TiO_2_ NPs, individually or in combination with 100 µM Cu and 100 µM Cd (the bars with various colors) based on Tukey′s test (*p* < 0.05).

The effects of TiO_2_ NPs and EBL concentrations on nonenzymatic activity (flavonol, tocopherol, and total phenolics) in the bamboo species revealed a significant difference between the co-application of 24-epibrassinolide and titanium oxide nanoparticles with Cu and Cd (*p* < 0.001). According to the results, the combination of TiO_2_-HMs and EBL- HMs significantly increased nonenzymatic antioxidant activities in our bamboo species. However, the greatest increase in nonenzymatic activity under heavy metal stress was related to the combination of TiO_2_ –EBL with Cu and TiO_2_–EBL with Cd, with a 1.55-fold and 1.51-fold enhancement in flavonols, 1.53-fold and 1.51-fold enhancement in tocopherols, and 1.68-fold and 1.58-fold increase in total phenolics, respectively, in comparison with the control treatment (Figure 3). Conversely, the concentrations of 100 µM Cu and 100 µM Cd clearly reduced nonantioxidant activity, as demonstrated by a 21% and 23% reduction in flavonols, 12% and 24% reduction in tocopherols, and 34% and 28% reduction in total phenolics, respectively, in comparison with the control treatment. We suggest that the combination of TiO_2_ and EBL has a positive impact on the reduction in heavy metal toxicity by stimulating nonenzymatic antioxidant activities (flavonols, tocopherols, and total phenolics).

### 3.4. 24-Epibrassinolide and Titanium Oxide Nanoparticles Improve Photosynthetic Pigments and Fluorescence Parameters in Bamboo Species under Cu and Cd Toxicity

**Figure 4:**
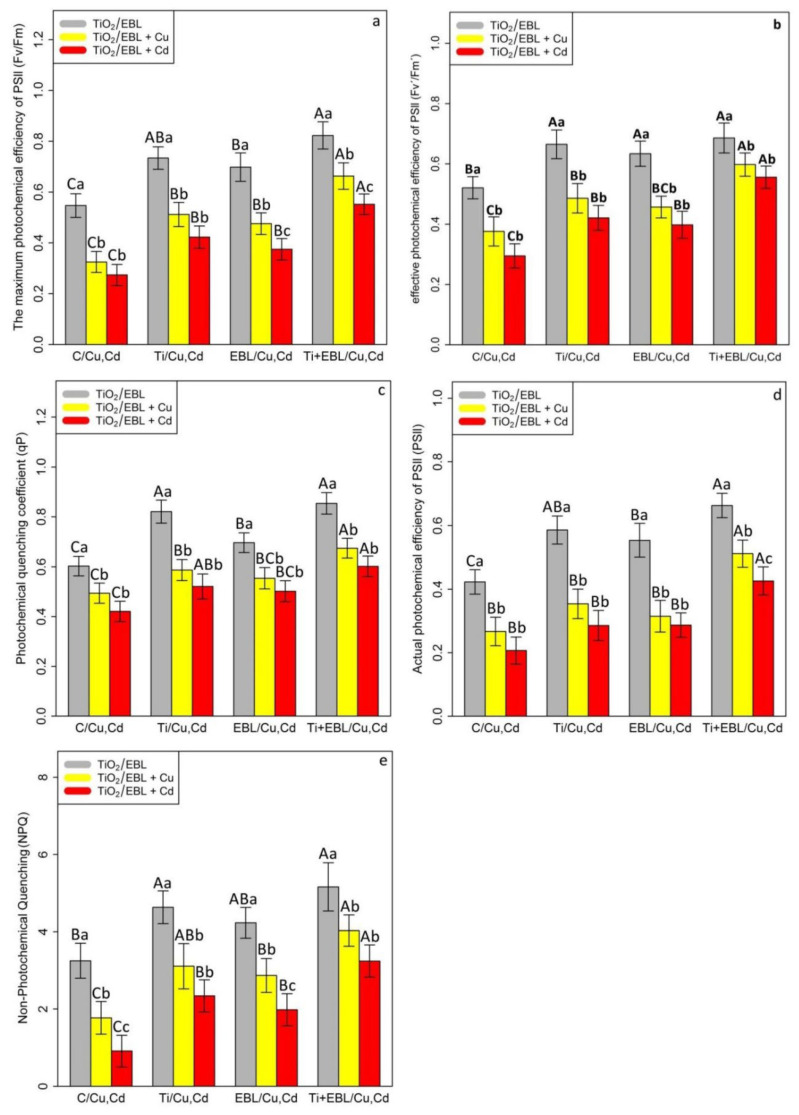
The effect of the co-application of 24-epibrassinolide and titanium oxide nanoparticles individually and combined on fluorescence parameters, including the maximum photochemical efficiency of PSll (Fv/Fm) (**a**), effective photochemical efficiency of PSll (Fv′/Fm′) (**b**), photochemical quenching coefficient (qP) (**c**), actual photochemical efficiency of PSll (φPSll) (**d**), and nonphotochemical quenching (NPQ) (**e**) in bamboo species (*Pleioblastus pygmaeus*) with 100 μM Cu and 100 μM Cd. In this study, 1-year-old branches of *P. pygmaeus* were used as plant treatments together with 100 µM TiO_2_ NPs and 10^−8^ M 24-epibrassinolide individually and in combination with 100 µM Cu and 100 µM Cd using four replications. Planting of the treated bamboo was performed in an Air Tech inoculation hood with fluorescent white lamps and ultraviolet light (wavelengths of 10–400 nm) at 15 °C and 30 °C. The bamboo plants were constantly exposed to excess heavy metals for three weeks. Sampling for the measurement of fluorescence parameters (**a**–**e**) was conducted after three weeks of plant exposure to the co-application of 24-epibrassinolide and titanium oxide nanoparticles under 100 µM Cu and 100 µM Cd. The capital letters (^A–C^) indicate significant differences between treatments of control (C), titanium (Ti), 24-epibrassinolide (EBL), and 24-epibrassinolide involving individual or combined application of titanium oxide nanoparticles (EBL–TiO_2_ NPs) under 100 μM Cu and 100 μM Cd (the bars with similar colors), while the lowercase letters (^a–c^) denote statistically significant differences at each concentration of the co-application of EBL and TiO_2_ NPs individually or in combination with 100 µM Cu and 100 µM Cd (the bars with various colors) based on Tukey′s test (*p* < 0.05).

Photosynthetic pigments and fluorescence parameters are important indices in the evaluation of photosynthetic efficiency in different species of plants under stress conditions. The indicators of plant photosynthesis performance, including photosynthetic pigments (Chl a, Chl b, and total Chl, as well carotenoid contents), and fluorescence indices, including the maximum photochemical efficiency of PSll (Fv/Fm), photochemical quenching coefficient (qP), effective photochemical efficiency of PSll (Fv′/Fm′), actual photochemical efficiency of PSll (φPSll), and nonphotochemical quenching (NPQ), were measured. We found a significant difference between the co-application of 24-epibrassinolide and titanium oxide nanoparticles with Cu and Cd (*p* < 0.001). Based on the results, the levels of TiO_2_NPs and EBL alone and in combination with heavy metals (Cu and Cd) could increase photosynthetic pigments in bamboo under Cu and Cd. However, the greatest increase in photosynthetic pigments under Cu and Cd was attributed to the co-application of TiO_2_–EBL with Cu and the co-application of TiO_2_–EBL with Cd, which resulted in 21% and 17% increases in Chl a, 85% and 83% increases in Chl b, 42% and 38% increases in total Chl, and 46% and 39% increases in carotenoid, respectively, in comparison with their control treatments (Table 2). Conversely, the measurement of the fluorescence parameters demonstrated a significant difference between the combination of TiO_2_–EBL and Cu and Cd (*p* < 0.001). The data analysis revealed similar results, e.g., in the Chl and carotenoid contents and in the measurement of fluorescence parameters. Therefore, the greatest increase in fluorescence parameters was related to the combination of TiO_2_ and EBL, which resulted in a 50% increase in the maximum photochemical efficiency of PSll (Fv/Fm), 41% increase in the photochemical quenching coefficient (qP), 54% increase in the effective photochemical efficiency of PSll (Fv′/Fm′), 56% increase in the actual photochemical efficiency of PSll (φPSll), and 58% increase in nonphotochemical quenching (NPQ) in comparison with their control treatments. We suggest that the combination of TiO_2_ NPs and EBL has a strong ability to increase photosynthesis parameters in plants exposed to heavy metal stress (Cu and Cd) (Figure 4).

### 3.5. 24-Epibrasinolide and Titanium Oxide Nanoparticles Reduce Heavy Metal Accumulation in Bamboo Leaves, Stems, and Roots

The decrease in metal accumulation in different types of plants is one of the main mechanisms responsible for the reduction of metal toxicity and increase in plant resistance when exposed to oxidative stress. Our results showed that TiO_2_ and EBL had a positive impact on the reduction in heavy metal concentrations in bamboo species; therefore, TiO_2_ and EBL alone or in combination significantly reduced heavy metal accumulation in leaves, stems, and roots (Table 3). This phenomenon is related to the role of TiO_2_ as a physical barrier that leads to a reduction in metal translocation from roots to aerial parts. The roots in plants are typically the first contact points where exposure to heavy metals occurs. Therefore, root physical traits are extremely decisive in limiting metal entry into plants. The root-based cellular layers comprised of epiblema, endodermis, and exodermis form apoplastic barriers in the roots, which can restrict heavy metal uptake by plants. We hypothesized that TiO_2_ NPs, through strengthening the apoplastic barriers in the roots and enhancing their impermeability, can significantly diminish the uptake of heavy metals. On the other hand, TiO_2_ NPs with high adsorption capacity act as an efficient binder of metal ions. Hence, TiO_2_ NPs can restrain the movement of heavy metals within the extracellular or intercellular parts of roots, thereby restricting heavy metal translocation from the root to shoot. TiO_2_ NPs may also have the ability to influence the expansion of the epidermal layer in plants, preventing heavy metal accumulation in nonphotosynthetic tissues by providing additional physical resistance. Conversely, we indicated that EBL, a hormone that is involved in plant growth regulation alone and in combination with TiO_2_, plays a positive role in the stimulation of antioxidant activity, which can scavenge ROS components in plant organs and inhibit plant oxidative stress caused by heavy metal toxicity. As shown in Table 3, the combination of TiO_2_–EBL with heavy metals showed the greatest reduction in heavy metal accumulation in the leaves, stems, and roots of bamboo species (Table 3). We suggest that TiO_2_ and EBL can reduce heavy metal contents in plant leaves, stems, and roots, thus demonstrating that the combination of TiO_2_ and EBL has the greatest impact on the decrease in metal toxicity.

**Figure 5:**
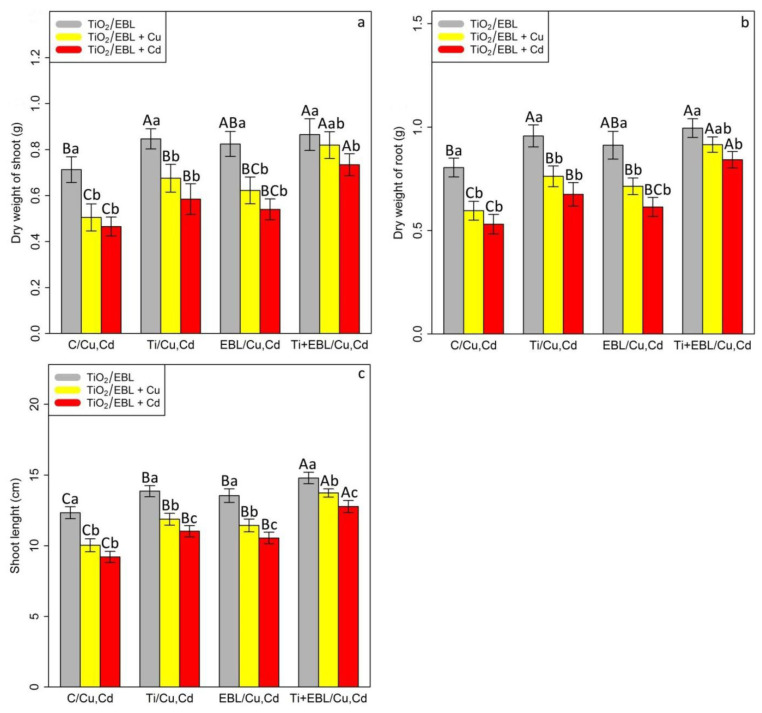
The impact of the co-application of 24-epibrassinolide and titanium oxide nanoparticles individually and combined on the dry weight of shoots (**a**), dry weight of roots (**b**), and shoot length (**c**) of bamboo species (*Pleioblastus pygmaeus*) with 100 μM Cu and 100 μM Cd. In this study, 1-year-old branches of *P. pygmaeus* were used as plant treatments together with 100 µM TiO_2_ NPs and 10^−8^ M 24-epibrassinolide individually and combined with 100 µM Cu and 100 µM Cd through four replications. Planting of the treated bamboo was performed in an Air Tech inoculation hood with fluorescent white lamps and ultraviolet light (wavelengths of 10–400 nm) at 15 °C and 30 °C. The bamboo plants were constantly exposed to excess heavy metals for three weeks. Sampling for the measurement of biomass indexes and plant growth (**a**–**c**) was conducted after three weeks of plant exposure to the co-application of 24-epibrassinolide and titanium oxide nanoparticles under 100 µM Cu and 100 µM Cd. The capital letters (^A–C^) indicate significant differences between treatments of control (C), titanium (Ti), 24-epibrassinolide (EBL), and 24-epibrassinolide involving individual or combined application of titanium oxide nanoparticles (EBL–TiO_2_ NPs) under 100 μM Cu and 100 μM Cd (the bars with similar colors), while the lowercase letters (^a–c^) denote statistically significant differences at each concentration of the co-application of EBL and TiO_2_ NPs individually or in combination with 100 µM Cu and 100 µM Cd (the bars with various colors) based on Tukey′s test (*p* < 0.05).

To evaluate the plant growth rate under Cu and Cd toxicity, the plant biomass, including root and shoot dry weight, as well as the length of shoots, were measured. A significant difference was found for the co-application of TiO_2_–EBL and heavy metals (*p* < 0.001) (Figure 5). Therefore, TiO_2_ and EBL individually and in combination significantly increased plant growth and biomass under stressful conditions. Based on this result, the greatest increase in plant biomass and growth under heavy metal exposure was related to the combination of TiO_2_ and EBL, which resulted in a 21% increase in the dry weight of shoots, a 23% increase in the dry weight of roots, and a 19% increase in the length of shoots in comparison with their control treatments (Table 4). Conversely, the measurements showed that the lowest plant growth was recorded under 100 µM Cu and 100 µM Cd, which resulted in 0.5 g and 0.46 g dry weights of shoots, 0.59 g and 0.53 g dry weights of roots, and 10.04 cm and 9.21 cm shoot lengths, respectively (Table 4).

The translocation factor (TF) is one of the main mechanisms used to evaluate the remediation efficiency of heavy metals in plant organs and the reduction of toxicity in plants under heavy metal stress. Therefore, it is calculated according to differences in the accumulation of Cu and Cd in shoots and roots, and it serves as an important factor in increasing plant tolerance to toxicity. In the present study, the addition of 24-epibrassinolide and titanium oxide nanoparticles significantly reduced Cu and Cd translocation from roots to shoots, which led to a reduction in toxicity by limiting metal accumulation in the plant aerial organs. Therefore, according to the results, the co-application of 24-epibrassinolide and titanium oxide nanoparticles in combination with heavy metals (Cu and Cd) resulted in a low level of translocation factor, which could reduce metal toxicity in the aerial parts of bamboo plants (Table 5). Additionally, the result showed that the co-application of EBL and TiO_2_ NPs significantly reduced Cu and Cd concentration in the leaves (*p* < 0.001), implicating the positive role of EBL–TiO_2_NPs in the reduction of heavy metal toxicity in the aerial parts of the bamboo plant (Table 5). Conversely, the calculation of the tolerance indices in shoots and roots revealed a significant difference between the co-application of TiO_2_ NPs and EBL alone under Cu and Cd (*p* < 0.001). Therefore, the levels of TiO_2_ NPs and EBL indicated an increase in shoot and root tolerance under heavy metal stress, which was obtained by the amelioration mechanism of the co-application of TiO_2_ and EBL against heavy metal toxicity, such as the stimulation of antioxidant activity and the increase in plant biomass. We suggest that TiO_2_ NP and EBL concentrations alone increase plant tolerance under metal stress; however, the most positive effect was more pronounced with the co-application of TiO_2_ NPs and EBL under Cu and Cd toxicity (Table 5).

## 4. Discussion

Titanium, as a form of TiO_2_, has the ability to alter the bioavailability and behavior of metals in the environment [45]. The impact of TiO_2_ NPs on increasing antioxidant activity and plant growth has been reported in several studies [13,14,46]. This increase can be attributed to the inductive role of TiO_2_ NPs in enhancing signaling associated with the activation of antioxidant enzyme activity [13]. This finding is consistent with the reported results in the present study. Therefore, our results demonstrated that the individual levels of TiO_2_ NPs could increase antioxidant and nonantioxidant activity in bamboo plants under certain Cu and Cd levels. Conversely, the level of EBL regulates plant stress by stimulating antioxidant activity [47]. The increasing capacity of antioxidant activity based on the levels of EBL in plants under stress has been reported in many studies [48,49,50]. EBL seems to play a main role in the activation of genes responsible for antioxidants by stimulating the expression of genes responsible for SOD, CAT, and APX in plants exposed to heavy metal stress [51]. Hence, it is interesting to note that EBL has the ability to ameliorate oxidative stress caused by metals, which has previously been reported for many plant species, such as *Brassica juncea* [20], *Cicer arietinum* [52], and *Raphanus sativa* [53]. The main reason can be attributed to the role of BR signaling kinase (BSK 1) in the stimulation of salicylic acid levels against oxidative damage [54]. In our studies, the application of TiO_2_ and EBL individually and in combination enhanced antioxidant enzyme activities, including SOD, POD, CAT, GR, APX, and PAL. However, the combination of TiO_2_ NP and EBL was more effective in increasing antioxidant levels than TiO_2_ NPs and EBL alone. Phenolic compounds, as nonenzymatic antioxidant activities, alleviate the negative effect of reactive oxygen radicals and have a strong ability to chelate metals [55,56]. There seems to be a relationship between enhancing phenylalanine ammonia-lyase (PAL) and the total phenolic compound, and it has been reported that PAL is a key enzyme responsible for the activation of the synthesis of phenolic compounds under stress [57,58]. This phenomenon has been reported in some studies on the reduction of Cu and Cd toxicity [55,59]. Our results demonstrated that the application of TiO_2_ NPs and EBL individually and in combination increased nonantioxidant activity (total phenolics, flavonols, and tocopherols) under Cu and Cd toxicity. This phenomenon could be related to PAL gene transcript levels as well as increasing PAL activity in response to EBL levels under heavy metal stress, which ultimately reduces ROS compounds by synthesizing phenolic compounds.

When antioxidant activity increases, the plant experiences cellular injuries (H_2_O_2_, MDA, and EL) [60]. It has been reported that TiO_2_ NPs induce certain stress-combative mechanisms, such as an improvement of the defense mechanism against ROS accumulation in plant intercellular space, which has been shown to attenuate H_2_O_2_ induction [60]. An increase in MDA content has been described as the initial stage of plant injury, which shows the rate of membrane lipid peroxidation [61]. This reveals the extent to which plants face this serious problem. Based on the present findings, we suggest that TiO_2_ NPs protect the plant cell membrane from ROS, a phenomenon that has been related to the role of TiO_2_ NPs in boosting antioxidant activity. Conversely, EBL has the ability to positively alter the membrane structure and membrane stability in plants exposed to stresses, such as heavy metals, which leads to a reduction in membrane lipid peroxidation [62]. In one study, the level of EBL was observed to diminish the concentrations of H_2_O_2_ and MDA (20–60% reductions) in plants under Pb stress [63]. In another study, EBL diminished the oxidative toxicity in cowpea under Cd by reducing lipid peroxidation, MDA content, and electrolytes [64,65]. In the present study, the level of EBL improved the ROS content and reduced the plant cell membrane under heavy metal toxicity. It is interesting to note that in this study, the application of TiO_2_ NPs and EBL individually and in combination decreased ROS and lipid peroxidation, including H_2_O_2_, O_2_^•−^, MDA, SP, and EL, in bamboo plants exposed to metal stress. One of the mechanisms underlying the amelioration of lipid peroxidation and ROS by EBL can be attributed to the increase in endogenous plant hormones that regulate plant growth, such as salicylic acid and ethylene, and the cross-talk between them. These mechanisms can improve plant tolerance under metal toxicity [66]. In this study, the co-application of TiO_2_ NPs and EBL was more efficient in reducing ROS compounds and ameliorating lipid peroxidation than TiO_2_ NPs and EBL alone.

Studies have indicated that TiO_2_ NPs enhance photosystem II in spinach by promoting oxygen evolution and energy transfer [67]. Additionally, TiO_2_ reduces Chl degradation and stimulates Chl biosynthesis, which can promote photosynthesis by stabilizing chlorophylls and carotenoids [60]. Chlorophylls are the most abundant component of the chloroplast and play an efficient role in the rate of photosynthesis [59]. The role of EBL in enhancing the cell number and photosynthetic pigment content (Chl a, Chl b, and carotenoids) has been demonstrated in some studies [68,69]. It seems that the increase in photosynthesis and Chl pigments by EBL is related to the stimulation activity of ribulose 1,5-bisphosphate carboxylase oxygenase as well as the increase in the Calvin cycle enzymes [20]. Therefore, the level of EBL with increasing carotenoids ameliorates photodamage during photosynthesis [70]. According to the above mechanisms, the co-application of EBL and Ti can improve photosynthesis and Chl pigment levels via Chl biosynthesis and reduce photodamage via the activation of the Calvin cycle enzymes in plants under heavy metal stress. Additionally, the results revealed a positive impact of the co-application of EBL and TiO_2_ NPs on fluorescence parameters, which showed an increase in the efficiency of fluorescence indices, including the maximum photochemical efficiency of PSll (Fv/Fm), photochemical quenching coefficient (qP), effective photochemical efficiency of PSll (Fv′/Fm′), actual photochemical efficiency of PSll (φPSll), and nonphotochemical quenching (NPQ). Therefore, we suggest that the co-application of EBL and TiO_2_ NPs can increase photosynthetic properties in plants exposed to heavy metals (Cu and Cd), and this phenomenon could be related to the increase in antioxidant activity and the reduction in heavy metal accumulation under metal toxicity stress.

**Figure 6:**
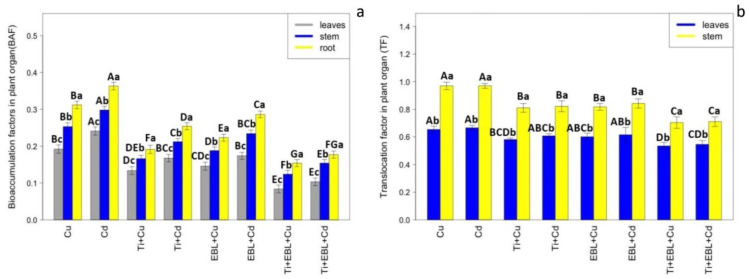
The impact of the co-application of 24-epibrassinolide and titanium oxide nanoparticles on BAF and TF: (**a**) the comparison between bioaccumulation of heavy metals in the root, stem, and leaves; (**b**) the comparison between translocation factor in the stem and the leaves. Bioaccumulation factor (BAF) is obtained by the difference between the concentrations of heavy metals in the leaf, stem, or root and concentrations of heavy metals in the medium, while the translocation factor (TF) is obtained by the difference between the concentration of heavy metals (Cu, Cd) in the leaves or stem of plants and the concentration of the heavy metals (Cu, Cd) in the roots of plants. The capital letters (^A–G^) indicate significant differences between treatments of control (C), titanium (Ti), 24-epibrassinolide (EBL), and 24-epibrassinolide involving individual or combined application of titanium oxide nanoparticles (EBL–TiO_2_ NPs) under 100 μM Cu and 100 μM Cd (the bars with similar colors), while the lowercase letters (^a–c^) denote statistically significant differences between leaves, stem, and root at each treatment (the bars with various colors) based on Tukey′s test (*p* < 0.05).

Titanium is known to be the most abundant transition element after iron, with a level of 1–578 mg kg^−1^ in different species of non-hyperaccumulator plants [71]. However, low mobility in the soil may impact its absorption by plants [72]. Our results showed that the root accumulation of TiO_2_ NPs was higher than that of stems and shoots, indicating that bamboo roots prefer to be storage organs of titanium, which has been reported in another study [73]. Therefore, the accumulation of titanium NPs led to the adsorption of heavy metals in roots, which, as a physical barrier, reduces metal translocation from roots to shoots. Conversely, EBL has the potential to reduce metal accumulation in plants by enhancing phytochelatin synthesis (PC) [48,49,50]. In a study on sugar beet, the levels of EBL reduced the absorption of heavy metals by plants by 50% [74,75,76]. It has also been reported [69] to reduce Cd accumulation in the roots, stems, and shoots of pea seedlings. Conversely, EBL levels reduce the accumulation of Cd by preserving ion homeostasis through the acceleration of calcium absorption [77,78]. Therefore, EBL leads to increased absorption of K^+^, mg^+^, and K^+^, which can be transported to the aerial parts of plants, such as leaves, and finally limit Cd and metal translocation from roots to shoots [79]. In the present study, the application of EBL and TiO_2_ could individually and in combination diminish the accumulation of heavy metals. These results are related to the role of EBL in preserving ion homeostasis, which can limit heavy metal uptake by coperception, which is associated with the role of TiO_2_ in the adsorption and absorption of heavy metals on the root surface. Similar to other transition metals, titanium, present in small fractions, absorbs to and accumulates in roots, as it is translocated through the xylem stream from roots to shoots [80]. The translocation of TiO_2_ NPs to aerial plant parts has been demonstrated [81,82]. As shown in Table 5, the co-application of TiO_2_ NPs and EBL significantly reduced metal translocation from roots to shoots, which is an important mechanism in increasing the tolerance of bamboo plants. However, the results showed that BAF in the roots was higher than in the stem and the leaves, which indicated that TiO_2_ NPs–EBL could effectively reduce BAF in the aerial parts of the bamboo plant. This could be explained by the mechanisms involved in the absorbance of Cu and Cd in the root surface by the co-application of TiO_2_ NPs and EBL. Therefore, we suggest that TiO_2_ NPs–EBL has an important role in the reduction of adsorption and uptake as well as the translocation of Cu and Cd to the aerial parts (leaves and stem). Thus, the application of TiO_2_ NPs–EBL can retain the heavy metals on the bamboo root surface. On the other hand, the results showed that the TF in the leaves was less than that in the stem, which was an indication that the heavy metals had been accumulated in the leaves less than in the stem (Figure 6).

Titanium is a useful element that can aid plant growth by increasing plant photosynthesis and enzymatic activity as well as increasing plant uptake of other nutrients [72]. One study reported that TiO_2_ can enhance the absorption rate of micro-and macronutrients, which could be the main factor in plant growth and biomass [83]. In our study, the role of TiO_2_ in increasing the plant biomass was related to an increase in nutrient absorbance by the plants and reduced toxicity in response to an increase in antioxidant activity. Many studies have reported that EBL increases plant growth under heavy metal stress [21,84,85]. The reduction in plant growth by heavy metal toxicity is related to the number of intercellular metal ions bound to the surface of the cell [86,87]. Our results showed that EBL could increase the plant biomass and plant growth under heavy metal toxicity, which could be related to the role of EBL in the reduction in intercellular metal ions, a phenomenon that has been confirmed in a study on A. *obliquus* [63]. However, the role of EBL in plant growth regulation during the stimulation of plant defense mechanisms can also be considered. Therefore, we hypothesized that EBL increased the plant growth under heavy metal stress by promoting antioxidant capacity. The increase in Chl as well as carotenoid contents in response to EBL has been confirmed in many studies [88,89,90,91]. Conversely, EBL has the ability to control cell division and elongation by regulating xyloglucan endotransglucosylase [92,93], thus demonstrating the positive role of EBL on plant growth and development, especially under stressful conditions. In the present research, the role of the co-application of TiO_2_ NPs and EBL in improving plant biomass and plant growth seemed to be related to the ameliorative mechanisms activated by the levels of both TiO_2_ NPs and EBL. The application of small-sized nanoparticles in the range of 1–100 nm is a new strategy to maintain plant growth and development under heavy metals and other abiotic stresses. However, the build-up of TiO_2_ NPs within the plant organs can have dual effects of growth promotion and suppression. Titanium dioxide nanoparticles (TiO_2_ NPs) lead to several beneficial outcomes on the physiological, morphological, and biochemical traits of some plant species, which has been indicated in some studies [94,95] as well as our present study. Conversely, some researchers have reported the detrimental impacts of high levels of TiO_2_ NPs on plants [96,97]. These implications might arise due to various environmental conditions, different plant species, and the applied levels [97,98]. Therefore, the safety/danger of TiO_2_ NPs for plants depends on a myriad of factors including size, concentration, method of treatment application, plant type and growth pattern, uptake amount by plants, cellular chemical properties, translocation rate, and reactivity of TiO_2_ NPs in various tissues, which determine NP interaction with a wide array of metabolic activities of the plants that can thus lead to their advantageous or toxic effects [99,100]. Additionally, the TiO_2_ NP surface area, their predisposition for accumulation in tissues, and their inherent reactivity are the possible reasons for their toxicological repercussions [101]. Therefore, there is a great need to take the aforementioned variables into careful consideration while applying TiO_2_ NPs in the agriculture and food industry, which can minimize health risk for humans. This finding revealed the effective role of the co-application of TiO_2_ NPs and EBL in comparison with TiO_2_ NPs and EBL individually.

## 5. Conclusions

Heavy metals are deemed a considerable environmental safety hazard with inhibitory impacts on plant growth due to the induction of excessive levels of ROS compounds, which causes oxidative stress in cells and tissues. The use of nanoparticles and phytohormones as two possible agents that mitigate the deleterious effects of heavy metals has been on the rise in the recent years. Thus, conducting extensive research using various plant species with distinct growth and morphological characteristics is needed. Based on our experimental results, the individual application of EBL as a phytohormone and TiO_2_ NPs contributed to the amelioration of toxicity in bamboo plants under excess Cu and Cd. However, the co-application of EBL and TiO_2_ demonstrated a greater effective influence on increasing plant tolerance under metal toxicity. Therefore, our results indicated that while Cu and Cd stress led to increased ROS production, causing injury to the plant membrane, by boosting oxidative activity, the co-application of EBL and TiO_2_ significantly reduced the ROS content and oxidative stress in the plants, resulting in an increase in the photosynthetic properties and an enhancement in the plant growth and development. Conversely, the co-application of EBL and TiO_2_ increased the plant tolerance under metal toxicity by reducing the heavy metal accumulation within the plant and restricting the metal translocation from the roots to the shoots. Overall, our study revealed the cellular-and tissue-level mechanisms involved in increasing bamboo plant tolerance to Cu and Cd toxicity through the integrated use of EBL and TiO_2_. This result requires further investigation with different plant species.

## Figures and Tables

**Table 1:** The treatment combinations of the experiment.

Treatments	Concentrations
Control	0
Cu	100 µM Cu
Cd	100 µM Cd
TiO_2_	100 µM TiO_2_
TiO_2_ + Cu	100 µM TiO_2_ + 100 µM Cu
TiO_2_ + Cd	100 µM TiO_2_ + 100 µM Cd
EBL	10^−8^ M EBL
EBL + Cu	10^−8^ M EBL + 100 µM Cu
EBL + Cd	10^−8^ M EBL + 100 µM Cd
TiO_2_ + EBL	100 µM TiO_2_ + 10^−8^ M EBL
TiO_2_ + EBL + Cu	100 µM TiO_2_ + 10^−8^ M EBL + 100 µM Cu
TiO_2_ + EBL + Cd	100 µM TiO_2_ + 10^−8^ M EBL + 100 µM Cd

**Table 2:** The effect of the co-application of 24-epibrassinolide and titanium oxide nanoparticles individually and combined on photosynthetic pigments (Chl a, Chl b, and total Chl, as well as carotenoid contents) in bamboo species (*Pleioblastus pygmaeus*) with 100 μM Cu and 100 μM Cd.

Treatments	Chl a (mg g ^−1^ F.w.)	Chl b (mg g ^−1^ F.w.)	Chl a + b (mg g ^−1^ F.w.)	Caratenoids (mg g ^−1^ F.w.)
Control	9.64 ± 0.57 ^Aa^	7.14 ± 1.24 ^Ba^	16.78 ± 1.76 ^Ba^	1.35 ± 0.23 ^Ba^
100 µM Cu	8.51 ± 0.54 ^Bab^	4.32 ± 1.75 ^Bb^	12.83 ± 2.03 ^Bb^	1.02 ± 0.11 ^Bab^
100 µM Cd	8.35 ± 0.60 ^Ab^	3.77 ± 1.16 ^Bb^	12.12 ± 1.57 ^Bb^	0.92 ± 0.12 ^Ab^
100 µM TiO_2_	10.71 ± 0.76 ^Aa^	10.14 ± 1.57 ^Aa^	22.35 ± 3.73 ^Aa^	1.82 ± 0.11 ^ABa^
100 µM TiO_2_ + 100 µM Cu	9.52 ± 0.67 ^ABab^	6.54 ± 0.95 ^ABb^	16.06 ± 1.62 ^Ab^	1.32 ± 0.33 ^ABb^
100 µM TiO_2_ + 100 µM Cd	9.17 ± 0.83 ^Ab^	4.43 ± 1.27 ^ABb^	13.60 ± 1.87 ^Bb^	0.92 ± 0.21 ^Ab^
10^−8^ M EBL	10.39 ± 0.54 ^Aa^	9.50 ± 1.01 ^ABa^	19.90 ± 1.35 ^Aa^	1.50 ± 0.24 ^Ba^
10^−8^ M EBL + 100 µM Cu	9.09 ± 0.77 ^ABab^	5.80 ± 0.97 ^ABb^	14.89 ± 1.66 ^Ab^	1.09 ± 0.09 ^ABa^
10^−8^ M EBL + 100 µM Cd	8.85 ± 0.67 ^Ab^	4.33 ± 1.31 ^ABb^	13.18 ± 1.97 ^Ab^	1.09 ± 0.25 ^Aa^
100 µM TiO_2_ + 10^−8^ M EBL	11.05 ± 1.22 ^ABa^	10.86 ± 0.82 ^Aa^	21.92 ± 1.97 ^Aa^	2.27 ± 0.27 ^Aa^
100 µM TiO_2_ + 10^−8^ M EBL + 100 µM Cu	10.23 ± 1.04 ^Aa^	8.01 ± 1.68 ^Ab^	18.24 ± 1.72 ^Aab^	1.48 ± 0.08 ^Ab^
100 µM TiO_2_ + 10^−8^ M EBL + 100 µM Cd	9.74 ± 0.98 ^ABa^	7.01 ± 1.44 ^Ab^	16.75 ± 2.08 ^Ab^	1.30 ± 0.12 ^Ab^

In this study, 1-year-old branches of *P. pygmaeus* were used as plant treatments together with 100 µM TiO_2_ NPs and 10^−8^ M 24-epibrassinolide individually and in combination with 100 µM Cu and 100 µM Cd using four replications. Planting of the treated bamboo was performed in an Air Tech inoculation hood with fluorescent white lamps and ultraviolet light (wavelengths of 10–400 nm) at 15 °C and 30 °C. The bamboo plants were constantly exposed to excess heavy metals for three weeks. Sampling for the measurement of photosynthesis pigments was conducted after three weeks of plant exposure to the co-application of 24-epibrassinolide and titanium oxide nanoparticles under 100 µM Cu and 100 µM Cd. The capital letters (^A,B^) indicate significant differences between treatments of control (C), titanium (Ti), 24-epibrassinolide (EBL), and 24-epibrassinolide with titanium oxide nanoparticles (EBL–TiO_2_ NPs) individually or in combination with 100 μM Cu as well as 100 μM Cd (the bars with similar colors), while the lowercase letters (^a,b^) denote statistically significant differences at each concentration of the co-application of EBL and TiO_2_ NPs individually or in combination with 100 µM Cu and 100 µM Cd (the bars with various colors) based on Tukey′s test (*p* < 0.05).

**Table 3:** The accumulation concentrations of titanium oxide nanoparticles and corresponding heavy metals (Cu and Cd) in bamboo shoots, stems, and roots.

Treatments	Heavy Metal in Leaves (µmol L^−1^)	TiO_2_ NP in Leaves (µmol L^−1^)	Heavy Metal in Stem (µmol L^−1^)	TiO_2_ NP in Stem (µmol L^−1^)	Heavy Metal in Root (µmol L^−1^)	TiO_2_ NP in Root (µmol L^−1^)
Control	0	0	0	0	0	0
100 µM Cu	19.22 ± 1.10 ^Ab^	0	25.3 ± 1.03 ^Ab^	0	31.2 ± 1.00 ^Ab^	0
100 µM Cd	24.20 ± 1.12 ^Aa^	0	29.8 ± 1.02 ^Aa^	0	36.3 ± 1.02 ^Aa^	0
100 µM TiO_2_	0	18.5 ± 0.90 ^Aa^	0	24.5 ± 0.95 ^Aa^	0	34.6 ± 1.04 ^Aa^
100 µM TiO_2_ + 100 µM Cu	13.42 ± 1.10 ^Bb^	14.3 ± 0.79 ^Ab^	16.6 ± 0.90 ^Cb^	18.5 ± 0.86 ^Ab^	19.1 ± 1.11 ^Cb^	28.5 ± 0.85 ^Ab^
100 µM TiO_2_ + 100 µM Cd	16.80 ± 0.97 ^Ba^	10.8 ± 0.87 ^Ac^	21.2 ± 0.86 ^Ca^	12.3 ± 0.86 ^Ac^	25.4 ± 0.95 ^Ca^	20.5 ± 0.90 ^Ac^
10^−8^ M EBL	0	0	0	0	0	0
10^−8^ M EBL + 100 µM Cu	14.60 ± 1.07 ^Bb^	0	18.8 ± 0.98 ^Bb^	0	22.3 ± 0.94 ^Bb^	0
10^−8^ M EBL +100 µM Cd	17.41 ± 0.97 ^Ba^	0	23.4 ± 0.94 ^Ba^	0	28.6 ± 0.90 ^Ba^	0
100 µM TiO_2_ + 10^−8^ M EBL	0	16.4 ± 0.77 ^Ba^	0	21.3 ± 0.95 ^Ba^	0	31.3 ± 0.94 ^Ba^
100 µM TiO_2_ + 10^−8^ M EBL +100 µM Cu	8.40 ± 1.07 ^Cb^	11.2 ± 0.87 ^Bb^	12.4 ± 1.02 ^Db^	15.4 ± 0.95 ^Bb^	15.4 ± 0.94 ^Db^	24.5 ± 1.02 ^Bb^
100 µM TiO_2_ + 10^−8^ M EBL +100 µM Cd	10.36 ± 0.99 ^Ca^	8.3 ± 0.90 ^Bc^	15.4 ± 0.97 ^Da^	10.2 ± 0.94 ^Bc^	17.7 ± 0.94 ^Da^	16.4 ± 0.86 ^Bc^

In this study, 1-year-old branches of *P. pygmaeus* were used as plant treatments together with 100 µM TiO_2_ NPs and 10^−8^ M 24-epibrassinolide individually and combined with 100 µM Cu and 100 µM Cd using four replications. The capital letters (^A–D^) indicate significant differences between treatments of control (C), titanium (Ti), 24-epibrassinolide (EBL), and 24-epibrassinolide with titanium oxide nanoparticles (EBL–TiO_2_ NPs) individually or in combination with 100 μM Cu as well as 100 μM Cd (the bars with similar colors), while the lowercase letters (^a–c^) denote statistically significant differences at each concentration of the co-application of EBL and TiO_2_ NPs individually or in combination with 100 µM Cu and 100 µM Cd (the bars with various colors) based on Tukey’s test (*p* < 0.05). 2–6 24-Epibrasinolide and titanium oxide nanoparticles increase plant biomass indices (root and shoot dry weight) and plant growth (length of shoot) in bamboo species under Cu and Cd toxicity.

**Table 4:** The changes in bamboo biomass in root and shoot dry weight as well as shoot length with 24-epibrassinolide and titanium oxide nanoparticles individually or combined with 100 μM Cu and 100 μM Cd in comparison with the control treatment.

Treatments	Dry Weight of Shoot (%)	Dry Weight of Root (%)	Shoot Length (%)
100 µM Cu	29%↓	26%↓	18%↓
100 µM Cd	35%↓	34%↓	25%↓
100 µM TiO_2_	18%↑	19%↑	12%↑
100 µM TiO_2_ + 100 µM Cu	5%↓	5%↓	3%↓
100 µM TiO_2_ + 100 µM Cd	17%↓	16%↓	10%↓
10^−8^ M EBL	15%↑	13%↑	10%↑
10^−8^ M EBL + 100 µM Cu	12%↓	11%↓	7%↓
10^−8^ M EBL + 100 µM Cd	24%↓	23%↓	14%↓
100 µM TiO_2_ + 10^−8^ M EBL	21%↑	23%↑	19%↑
100 µM TiO_2_ + 10^−8^ M EBL +100 µM Cu	15%↑	13%↑	11%↑
100 µM TiO_2_ + 10^−8^ M EBL +100 µM Cd	3%↑	4%↑	3%↑

3–7 24-Epibrasinolide and titanium oxide nanoparticles reduce the translocation factor (TF) and bioaccumulation factor (BAF) and improve the tolerance index (TI) in roots and shoots of bamboo species. ↑ indicates increases and ↓ indicates decreases.

**Table 5:** Changes in the translocation factor and tolerance index of shoots and roots in response to 24-epibrassinolide and titanium oxide nanoparticles individually or in combination with 100 μM Cu and 100 μM Cd compared with the control treatment. Each data point is the mean ± SE of four replicates. The capital letters (^A–C^) indicate significant differences between treatments of control (C), titanium (Ti), 24-epibrassinolide (EBL), and 24-epibrassinolide involving individual or combined application of titanium oxide nanoparticles (EBL–TiO_2_ NPs) under 100 μM Cu and 100 μM Cd (the bars with similar colors), while the lowercase letters (^a–c^) denote statistically significant differences at each concentration of the co-application of EBL and TiO_2_ NPs individually or in combination with 100 µM Cu and 100 µM Cd (the bars with various colors) based on Tukey′s test (*p* < 0.05).

Treatment	Translocation Factor (Leaves)	Tolerance Index (Shoot)	Tolerance Index (Root)	Bioaccumulation Factor (Leaves)
Control	0.00 ± 0.00 ^Bb^	1.00 ± 0.00 ^Ba^	1.00 ± 0.00 ^Ba^	0.00 ± 0.00 ^Aa^
100 µM Cu	0.65 ± 0.02 ^Aa^	0.70 ± 0.06 ^Cb^	0.74 ± 0.09 ^Bb^	0.19 ± 0.01 ^Ab^
100 µM Cd	0.66 ± 0.01 ^Aa^	0.65 ± 0.05 ^Cb^	0.66 ± 0.09 ^Bb^	0.24 ± 0.01 ^Ac^
100 µM TiO_2_	0.52 ± 0.04 ^Ab^	1.19 ± 0.13 ^ABa^	1.23 ± 0.12 ^Aa^	0.00 ± 0.00 ^Aa^
100 µM TiO_2_ + 100 µM Cu	0.58 ± 0.01 ^BCa^	0.94 ± 0.07 ^ABb^	0.95 ± 0.11 ^ABb^	0.13 ± 0.01 ^Bb^
100 µM TiO_2_ + 100 µM Cd	0.60 ± 0.01 ^ABa^	0.80 ± 0.05 ^Bb^	0.84 ± 0.10 ^Bb^	0.16 ± 0.01 ^Bc^
10^−8^ M EBL	0.00 ± 0.00 ^Bb^	1.15 ± 0.09 ^ABa^	1.13 ± 0.13 ^ABa^	0.00 ± 0.00 ^Aa^
10^−8^ M EBL + 100 µM Cu	0.60 ± 0.02 ^Ba^	0.87 ± 0.08 ^BCb^	0.89 ± 0.09 ^ABb^	0.14 ± 0.01 ^Bb^
10^−8^ M EBL + 100 µM Cd	0.61 ± 0.05 ^Aa^	0.75 ± 0.01 ^Bb^	0.76 ± 0.09 ^Bb^	0.17 ± 0.00 ^Bc^
100 µM TiO_2_ + 10^−8^ M EBL	0.49 ± 0.01 ^Ab^	1.21 ± 0.08 ^Aa^	1.24 ± 0.12 ^Aa^	0.00 ± 0.00 ^Aa^
100 µM TiO_2_ + 10^−8^ M EBL + 100 µM Cu	0.53 ± 0.02 ^Cab^	1.1 ± 0.09 ^Aab^	1.09 ± 0.10 ^Aa^	0.08 ± 0.01 ^Cb^
100 µM TiO_2_ + 10^−8^ M EBL + 100 µM Cd	0.54 ± 0.02 ^Ba^	1.02 ± 0.04 ^Ab^	1.04 ± 0.08 ^Aa^	0.10 ± 0.01 ^Bc^

## Data Availability

The data presented in this study are available in article.
